# Global and Local Aging in Differently Stabilized Polypropylenes Exposed to Hot Chlorinated Water with and without Superimposed Mechanical-Environmental Loads

**DOI:** 10.3390/polym11071165

**Published:** 2019-07-08

**Authors:** Joerg Fischer, Reinhold W. Lang, Patrick R. Bradler, Paul J. Freudenthaler, Wolfgang Buchberger, Susan C. Mantell

**Affiliations:** 1 Institute of Polymeric Materials and Testing, Johannes Kepler University Linz, Altenberger Strasse 69, 4040 Linz, Austria; 2 Institute of Analytical Chemistry, Johannes Kepler University Linz, Altenberger Strasse 69, 4040 Linz, Austria; 3 Department of Mechanical Engineering, University of Minnesota, 111 Church Street SE, Minneapolis, MN 55455, USA

**Keywords:** polypropylene, stabilizer system, chlorinated water, aging, fatigue crack growth resistance, superimposed mechanical-environmental testing

## Abstract

The influence of chlorinated water on the global and local aging behavior of polypropylene (PP) was investigated for three differently stabilized PP grades consisting of the same PP base polymer. While one of the PP grades contained only a processing stabilizer (PP-S0), the other two were modified with a primary phenolic antioxidant (PP-S1) and a combination of a primary phenolic antioxidant and a hindered amine stabilizer (PP-S3). To study global aging effects, micro-sized specimens were pre-exposed to chlorinated water (5 mg/L free chlorine) at 60 °C for up to 750 h. Over the entire exposure period, significant material aging was detected by monitoring a continuous decrease in stabilizer content, oxidation induction temperature, mean molar mass, and mechanical strain at break. In terms of aging resistance and ultimate mechanical performance, PP-S1 was found to outperform the other two material formulations under these test conditions. Moreover, superimposed mechanical-environmental fatigue tests with cracked round bar specimens were carried out with the three PP grades in non-chlorinated (0 mg/L free chlorine) and chlorinated (5 mg/L free chlorine) water at 80 °C and 95 °C to study local crack tip aging effects. While the fatigue crack growth resistance substantially deteriorated for all three materials in chlorinated water, a significantly stronger effect was found for the higher temperature, with crack growth rates at a given stress intensity factor range in chlorinated water being ca. 30 to 50 times faster than in non-chlorinated water, depending on the material. Molar mass measurements of material samples taken from various positions of the tested CRB specimens provided clear evidence of enhanced local crack tip aging due to the chlorinated water environment.

## 1. Introduction

Polypropylene (PP) is a commonly used material for plastics pipes, specifically for pressurized domestic hot water piping with water temperatures up to 60 °C [1,2,3]. More recently, specifically molecularly designed and stabilized PP grades have become of interest for applications in modern polymer-based solar-thermal systems. This includes both structural components exposed to only moderate stress levels (e.g., assembly components and containments) but also highly stressed components such as pressurized pipes. Temperatures in such solar-thermal applications can reach maximum temperatures of 80 °C to 95 °C [4,5,6,7].

In both areas of applications, which include conventional hot water piping and solar-thermal systems, disinfected tap water is often used against pathogens to prevent the spread of waterborne diseases, with the type and concentration of disinfectant depending on the world region. The most widely utilized disinfectant is chlorine, which is applied as aqueous solution of chlorine gas or sodium hypochlorite [8,9,10]. For the disinfection of tap water, typically, chlorine contents of up to 5 mg/L free chlorine and a pH between 6.5 and 7.6 are recommended [9,10]. Moreover, to prevent the growth and spread of legionella, hot water temperatures of 60 °C are required [11].

Due to their oxidative nature, any such chlorine-based disinfectants may also significantly affect the aging behavior and, hence, the performance of polymeric materials [12,13,14,15,16,17,18,19,20,21,22,23,24,25,26,27,28,29,30]. For example, numerous reports exist on accelerated aging and on premature failures of hot water polyolefin pipes exposed to chlorinated water [21,26,27,28,29,30,31]. Moreover, it has been proposed in previous research that the simultaneous and superimposed influence of mechanical stresses and environmental media may be the prime cause for accelerated aging, as observed under service conditions [12,14,15,16,17,19,23,24]. Hence, specifically severe conditions of superimposed mechanical-environmental aging exist in regions of stress concentrations (e.g., notches) and the highly stressed regions at crack tips. In fact, for the latter case, the mechanism of enhanced local crack tip aging has been proposed to explain the observed increase in crack growth rates when materials were investigated under pure mechanical and under superimposed mechanical-environmental test conditions [32,33,34,35,36].

To counteract such accelerated aging, various types of stabilizer systems are typically incorporated in polymeric materials [13,20,25,37,38]. In this context, it is important to recognize that the applicability and effectivity of stabilizer systems not only depend on the environmental conditions but also on the molecular and morphological nature of the polymeric base material. Considering this complexity and the necessity for a manageable methodological approach to cover aspects of global and local aging, it is not surprising that only a limited amount of investigations exists on the effects of various stabilizer systems on the global and local aging behavior in polypropylene [4,5,6,7,12,13,14,15,16,17,19,20,39,40,41,42,43]. Nevertheless, regarding the methodological approach, novel test methods have been implemented in the laboratory of the Institute of Polymeric Materials and Testing at the Johannes Kepler University Linz to investigate such phenomena of pure environmental global aging (without stresses) and superimposed mechanical-environmental local aging in an efficient manner [4,5,6,7,12,13,14,15,17,20,40,42].

Hence, it is the objective of this paper to systematically investigate the prime mechanisms controlling global and local aging in differently stabilized polypropylenes under hot chlorinated water exposure. Therefore, three PP grades with different stabilizer packages were characterized in two test series. In one series, for studying global aging, micro-sized specimens were pre-exposed in hot chlorinated water and, subsequently, characterized in terms of various aging indicators such as stabilizer content, oxidation induction temperature, mean molar mass, and strain at break. The other test series, which aimed at the elucidation of superimposed mechanical-environmental aging phenomena and mechanisms on a more local crack tip scale, involved fracture mechanics experiments under cyclic loads of cracked round bar specimens immersed in hot non-chlorinated and chlorinated water while being tested. In this scenario, the kinetics of failure is taken as an aging indicator supplemented by mean molar mass and molar mass distribution analysis of material taken from the crack tip region.

## 2. Materials and Methods

### 2.1. Materials

**Figure 1:**
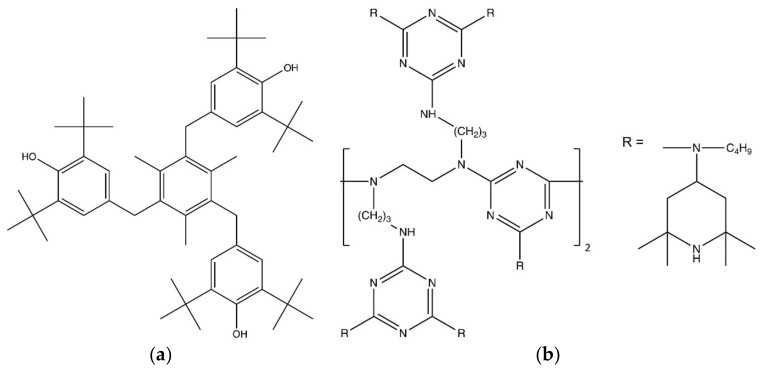
Chemical structure of the stabilizers. (**a**) Primary phenolic antioxidant (Irganox 1330). (**b**) Hindered amine stabilizer (Uvasorb HA88).

Experiments were conducted with three polypropylene (PP) model material grades, which differed in their stabilizer package (see Table 1). A PP homopolymer (Ineos Group, London, UK) with a high stiffness and low melt flow rate containing only a processing stabilization was used as reference material (PP-S0). For investigating the effect of the stabilizer system, this reference material was additionally stabilized by compounding with a primary phenolic antioxidant, on the one hand (PP-S1), and with a combination of a primary phenolic antioxidant and an alkyl radical scavenger, on the other hand (PP-S3). The commercially available sterically hindered phenol Irganox 1330 (BASF, Basel, Switzerland) was utilized as the primary antioxidant, and the commercially available oligomeric hindered amine stabilizer Uvasorb HA88 (3V Sigma, Bergamo, Italy) was used as the alkyl radical scavenger. Chemical structures of the stabilizers are provided in Figure 1. The materials are identical to those used in previous publications on the fracture and failure behavior of these materials [13,16].

### 2.2. Specimens

To study the global aging behavior, micro-sized specimens [13,44] with the dimensions 150.0 mm × 2.0 mm × 0.1 mm (length × width × thickness) were used. The specimens were planed from 2 mm thick injection molded (Victory 60, Engel, Schwertberg, Austria) plates with a 4-axis milling machine of the type EMCO Mill E600 (EMCO, Hallein, Austria).

For investigating superimposed mechanical-environmental local crack tip aging, cracked round bar (CRB) specimens [45] with a diameter of 14 mm were used and exposed to cyclic loads while being immersed in the various liquids at the pre-defined temperatures. The CRB specimens were machined from 15 mm thick compression molded plaques with a lathe of the type EMCO 14D (EMCO, Austria). Prior to the cyclic tests, the initial crack with a length of 1.5 mm was cut with a razor blade, which was mounted on the lathe.

### 2.3. Monitoring of Global Aging with Micro-Sized Specimens

A controlled water bath [12,13,20,22,46] was utilized for the pre-exposure of the micro-sized specimens. Specimens were immersed in chlorinated water with a chlorine content of 5 mg/L free chlorine and a pH of 7 at 60 °C for up to a total exposure time of 750 h. After 125 h, 250 h, 375 h, 500 h, and 750 h, respectively, six specimens each were removed for subsequent analysis and mechanical tensile tests.

High-pressure liquid chromatography (HPLC) coupled with the ultraviolet detector was conducted to analyze the consumption of the primary antioxidant and the hindered amine stabilizer. The experiments were performed using an HPLC apparatus of the type 1260 Infinity (Agilent, Santa Clara, CA, USA) with a Kinetex C18 separation column (Phenomenex, Torrance, CA, USA).

A differential thermal analysis (DTA) instrument of the type DSC 4000 (PerkinElmer, Waltham, MA, USA) was utilized to characterize the oxidation induction temperature (dynamic OIT) [47] and the PP melting peak as a function of exposure time. For the dynamic OIT measurements, a single heating step was performed between 23 °C and 300 °C with a heating rate of 10 K/min in a synthetic air environment. To obtain melting peaks that reflect an equivalent thermal crystallization history, samples were first heated (23–200 °C), then cooled (200–23 °C), and, lastly, heated again (23–200 °C), in all stages with a heating and cooling rate, respectively, of 10 K/min in a nitrogen environment. All tests were carried out with samples with a weight of about 5 mg, which were positioned in a perforated aluminum pan (PerkinElmer, Waltham, MA, USA).

Gel permeation chromatography (GPC) was conducted with a high-temperature gel permeation chromatograph (PolymerChar, València, Spain) to characterize the mean weight average molar mass (M_w_) and the molar mass distribution of the non-exposed and pre-exposed specimens. The chromatograph was equipped with an IR 5 detector, and it was calibrated with a polypropylene standard. Samples with a weight of about 2 mg were cut from the specimens and were dissolved in trichlorobenzene (VWR International, Radnor, PA, USA). As a flow marker, heptane (VWR International, Radnor, PA, USA) was added.

To provide data as performance indicators for global aging, tensile tests were carried out with six micro-sized specimens for each exposure time to determine the mean values for strain at break. Tests were conducted at 23 °C with a 100 N load cell mounted on a universal testing machine of the type Instron 4202 (Instron, Norwood, MA, USA). The gauge length was 20 mm, and the test speed was 50 mm/min.

### 2.4. Fatigue Crack Growth Behavior and Monitoring of Local Aging under Superimposed Mechanical-Environmental Conditions Tested with Cracked Round Bar (CRB) Specimens

Fatigue crack growth (FCG) experiments with CRB specimens were performed on the tension-torsion electro-dynamic testing machine of the type ElectroPuls E10000 (Instron, Norwood, MA, USA), which was equipped with a self-developed environmental containment to allow for superimposed mechanical-environmental tests. Environmental conditions were controlled via a chlorine control unit together with a temperature control unit, with chlorine contents set to 0 mg/L free chlorine (non-chlorinated water) and 5 mg/L free chlorine (chlorinated water), respectively, with both holding a pH of 7 and temperatures of 80 °C and 95 °C. The test system was equipped with an optical crack length measurement device, which was connected with a self-designed software, and which was used for quasi-automatic in-situ optical crack length measurements. Further details on the testing system, the environmental containment, and the corresponding control units along with information on the in-situ crack length measurement system are described elsewhere [14,15].

The CRB specimens, which were stored in laboratory air and not exposed to the environmental conditions prior to testing, were loaded under sinusoidal force control with maximum applied forces depending on the PP grade and the environmental test conditions to allow for reasonable testing times (maximum of 82 h). The test frequency was 10 Hz and the R-ratio (ratio between minimum and maximum force) was 0.1. Experimental results are presented as FCG curves, which are plotted as double-logarithmic graphs of the crack growth rate (da/dN) as a function of the stress intensity factor range (ΔK). Crack growth rate values were calculated from mean crack length data using a secant method and following a procedure described by Fischer et al. [14,15]. The values for the stress intensity factor range were calculated by using CRB specimen-specific equations [12,15].

**Figure 2:**
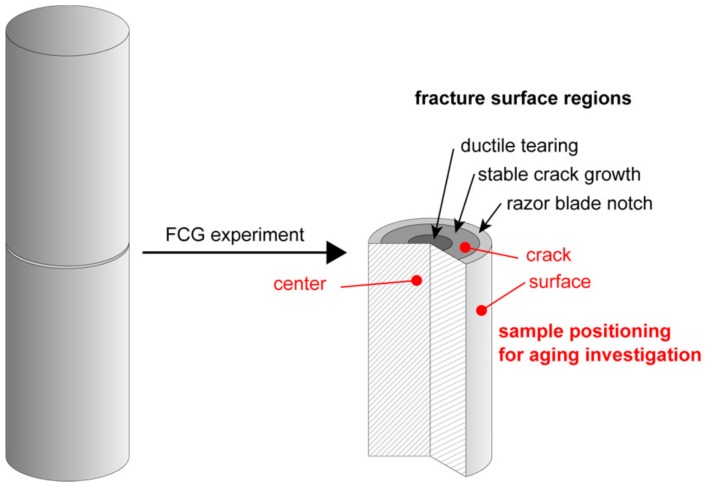
Schematic of the CRB specimen (**left**) before and (**right**) after fatigue crack growth (FCG) experiments indicating the three sample positions (i.e., center, surface, and crack) for the investigation of the corresponding local aging states.

To characterize the local aging states at various positions of a CRB specimen after an FCG experiment had been completed and the CRB specimen was fractured, samples were taken to determine the mean molar mass (M_w_) and molar mass distribution from three different positions, which are depicted in Figure 2. Two of these positions were remote from the crack tip, with one corresponding to a center volume element of the CRB specimen, which is not direct and, hence, least exposed to the environmental medium (“center” position in Figure 2). The second position corresponds to a volume element at the outer surface of the CRB specimen, which is most exposed to the environmental medium since it is in direct contact with the medium over the entire test duration (“surface” position in Figure 2). The third sample position of specific interest to characterize local crack tip aging is located directly on the fracture surface, and samples were taken from the stable crack growth region of the fracture surfaces of the CRB specimens tested and ultimately fractured (“crack” position in Figure 2).

## 3. Results

In the following, the results of global aging during chlorinated water exposure at 60 °C of micro-sized specimens without superimposed stresses will be presented and discussed. Subsequently, the results of the superimposed mechanical-environmental FCG experiments in various environments and at different temperatures together with results of investigations regarding various local aging states as they develop in CRB specimens at three different positions will be described (see Figure 2).

### 3.1. Effect of Chlorinated Water on Global Aging Behavior of Micro-Sized Specimens

**Figure 3:**
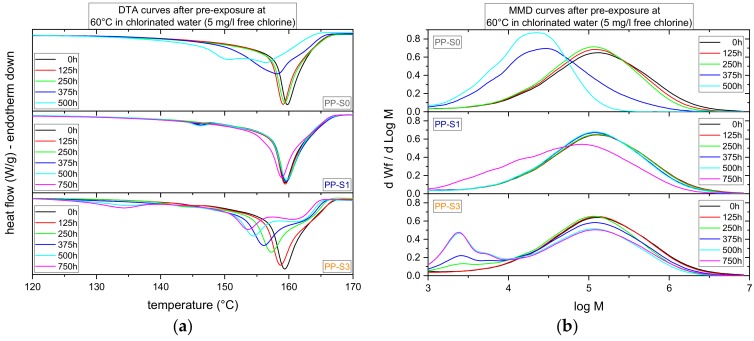
(**a**) Differential thermal analysis (DTA) curves indicating the melting peaks in the second heating step and (**b**) molar mass distribution (MMD) curves for various exposure times of micro-sized specimens to chlorinated water (5 mg/L free chlorine) at 60 °C for the three polypropylene (PP) grades.

Results for the morphological and molecular material aging states of micro-sized specimens of the three PP grades exposed to chlorinated water (5 mg/L free chlorine) at 60 °C for up to 750 h are depicted in Figure 3a,b in terms of differential thermal analysis (DTA) curves and molar mass distribution (MMD) curves, respectively. Starting with PP-S0, the PP-grade without long-term stabilization, the main melting peak temperatures from the second heating step, and the number of long chain molecules in the MMD curve continuously decrease with increasing exposure time. While, at up to 250 h of exposure, these changes are quite moderate, more significant changes appear after 375 h and 500 h. For PP-S1, stabilized with a primary antioxidant, up to an exposure time of 500 h, only minor variations in melting peak and molar mass distribution were detected. Significant shifts in melting peak temperature and molar mass distribution are apparent after 750 h of exposure. Therefore, the positive effect of the addition of a primary antioxidant is corroborated. Turning to PP-S3 with a stabilization package of a primary antioxidant and a hindered amine stabilizer, an entirely different evolvement of the aging material states was found. In fact, in this material aging seems to take place continuously right from the start of exposure, with a bimodal and, subsequently, multimodal MMD characteristic developing after 250 h of exposure. Simultaneously, a bimodal melting peak appears in the DTA curves, which indicates a morphological inhomogeneity, to some extent. When comparing the three material grades in terms of morphological and molecular global aging kinetics, clearly PP-S1 exhibits the best behavior with PP-S3 displaying even more rapid aging compared to PP-S0, which contained no long-term stabilization. From these findings, it may be concluded that the addition of a hindered amine stabilizer when combined with a conventional primary phenolic antioxidant may, in fact, lead to an antagonistic rather than a synergistic aging characteristic, which may lead to faster aging than without any stabilization at all.

**Figure 4:**
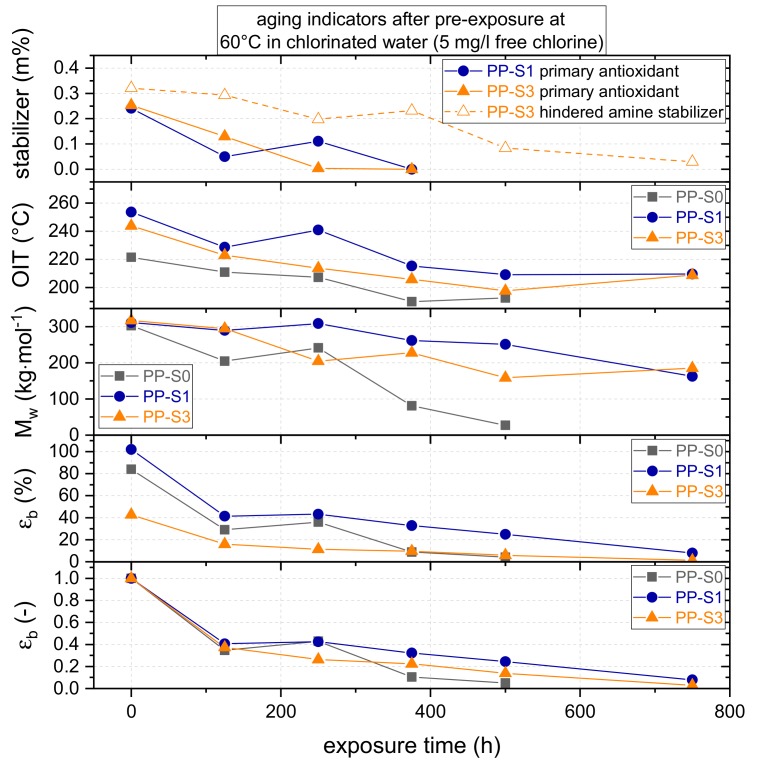
Stabilizer content (primary antioxidants and hindered amine stabilizer), dynamic oxidation induction temperature (OIT), mean weight average molar mass (M_w_), strain at break (ε_b_), and normalized strain at break as a function of exposure time of micro-sized specimens for the three PP grades.

To compare the global aging behavior of the three materials as a function of exposure time, a summary of the various aging indicators, as defined in Section 2.3, is provided in Figure 4. Starting with the progress in stabilizer loss for the two additionally stabilized PP grades PP-S1 and PP-S3, the content of primary antioxidants decreases substantially with increasing exposure time, which results in total antioxidant loss (i.e., 0 m%) after 375 h in both materials. The hindered amine stabilizer in PP-S3 was consumed/extracted continuously over the entire exposure time with the final content of 0.03 m% after 750 h.

Regarding the presence of stabilizers, when comparing the oxidation induction temperatures (dynamic OIT), the effect of the additionally introduced stabilizers is evident from the significantly higher dynamic OIT values for PP-S1 and PP-S3 initially and over the exposure time. With 254 °C and 244 °C, respectively, the initial dynamic OIT values for PP-S1 and PP-S3 are more than 20 °C higher than the value for PP-S0 with 221 °C. However, despite the higher amount of the total stabilizer content in PP-S3, PP-S1 showed a 10 °C higher initial dynamic OIT value, which, again, indicates some antagonistic effect related to the presence of the hindered amine stabilizer. Looking at the aging-dependent evolution of the dynamic OIT data, all PP grades exhibited a decrease over the experimental exposure time with final values of 193 °C after 500 h for PP-S0, and with 210 °C and 209 °C after 750 h, respectively, for PP-S1 and PP-S3. Thus, the drop in dynamic OIT values over the exposure time amounted to some 30 °C to 45 °C depending on the material formulation. Since PP-S0 contained no long-term stabilization, the continuous decrease in dynamic OIT data with exposure time cannot be related to stabilizer loss or consumption during the exposure but may be a result of the polymer degradation process for which evidence was already provided in Figure 3. By comparison, the decrease in dynamic OIT for PP-S1 and PP-S3 is in good agreement with the stabilizer loss data discussed above, which does not exclude some contribution from molecular degradation in these cases. Yet, from an overall perspective, PP-S1, which is the PP grade stabilized with a primary antioxidant, exhibited the highest dynamic OIT values over the entire exposure time.

As to molecular aging indicators, all PP grades were affected by the exposure to hot chlorinated water, which led to continuously decreasing mean weight average molar mass (M_w_) values. However, this includes different kinetics depending on the stabilization. The initial M_w_ values for PP-S0, PP-S1, and PP-S3 with 303 kg/mol, 311 kg/mol, and 317 kg/mol, respectively, were rather similar. With continuous chlorinated water exposure, especially PP-S0 (no long-term stabilization) exhibited a pronounced drop in M_w_ after 175 h, which indicates significant chemical aging and material degradation after such a short exposure time. Further hot chlorinated water exposure caused additional aging, and, after 500 h, a final M_w_ value of about 27 kg/mol was found, which represents a value less than 10% of the initial M_w_ value of PP-S0. Conversely, the M_w_ values of PP-S1 and PP-S3 after 750 h of exposure dropped to about 163 kg/mol and 185 kg/mol, respectively, which represents an M_w_ deterioration of approximately 50%. Again, when comparing all three PP grades, PP-S1 over most of the exposure time shows the lowest tendency for molecular degradation, which reaches a rather equivalent state of molecular degradation to PP-S3 after the longest exposure time of 750 h. It should be noted, however, that these equivalences in the mean molar mass between PP-S1 and PP-S3 after 750 h of exposure are a result of entirely different molar mass distributions, which was discussed with reference to Figure 3b.

Moving now to the strain at break (ε_b_) data as the material performance indicator for global aging, it is apparent from Figure 4 that, under unexposed conditions, PP-S3 with 43% reveals a significantly lower ε_b_ value than PP-S0 and PP-S1 with 84% and 102%, respectively. This is somewhat surprising, considering that, in the unexposed state, all three material grades exhibit quite comparable M_w_ values and MMD as well as equivalent degrees of crystallinity and melting temperatures. Apparently, the addition of the specific hindered amine stabilizer type used in PP-S3 even acts antagonistic with regard to the material performance in the unexposed material state. According to other investigations, this is due to the primary phenolic antioxidant becoming increasingly acidic, which interacts with the alkaline environment of the hindered amine stabilizer. The resulting antagonistic acid/base mechanism, which involves the consumption of phenol by the activated hindered amine stabilizer, is also discussed in literature [48,49,50]. Moreover, this may be related to the insufficient compatibility of the stabilizer with the base polymer, which leads to the stabilizer aggregates at inter-spherulitic boundaries acting as inherent defects.

For the dependence of the strain at break (ε_b_) data on the exposure time, a normalized presentation with strain at break at given exposure times divided by the initial strain at break without exposure is depicted in Figure 4. Considering the initial values for ε_b_, after 125 h of exposure for all three PP grades, ε_b_ drops to less than a half of the initial values. However, it remains unclear to which extent this is caused by physical aging (post/re-crystallization) vs. chemical aging (molecular degradation). A further drop in strain at break occurs for PP-S0 after 375 h, whereas the ε_b_ values for the other two PP grades show a more continuous decrease with increasing exposure time. Quasi-total embrittlement, with ε_b_ values remaining below overall specimen yielding, is reached for PP-S0, PP-S1, and PP-S3 after 500 h, 750 h, and 750 h, respectively. In terms of the strain at break, PP-S1 containing just the primary antioxidant outperforms PP-S0 (no stabilization) and PP-S3 (primary antioxidant plus a hindered amine stabilizer) over the entire exposure time.

### 3.2. Effect of Chlorinated Water on the Fatigue Crack Growth Resistance and Local Aging Behavior in Cracked Round Bar (CRB) Specimens

**Figure 5:**
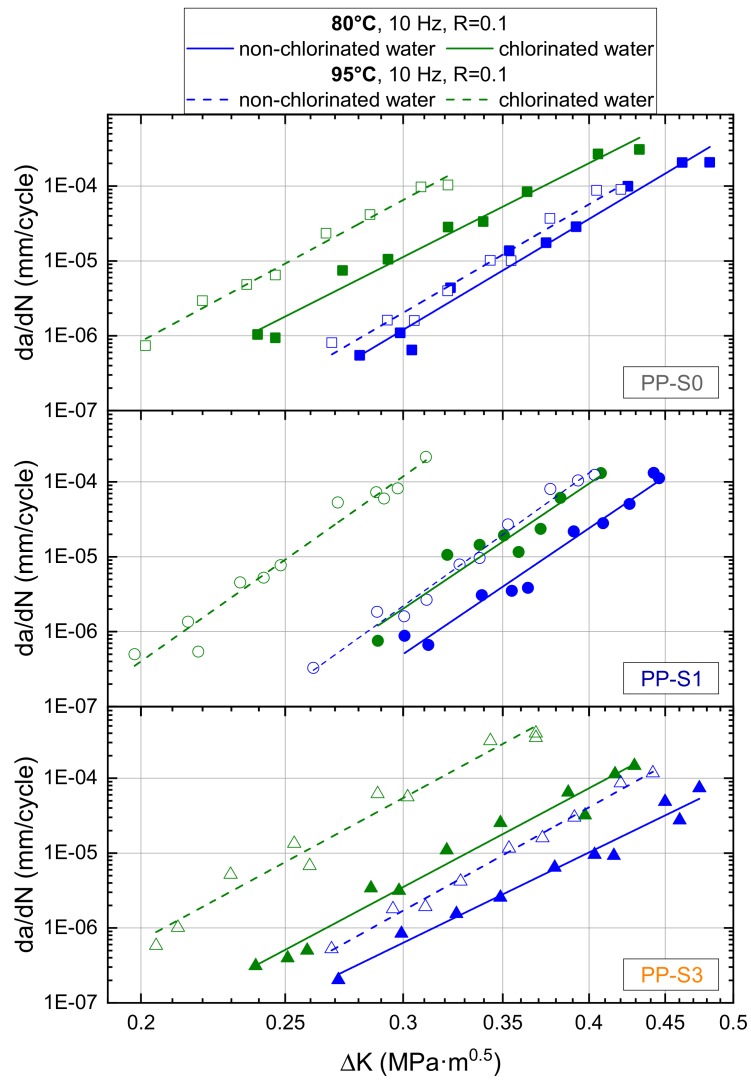
Fatigue crack growth (FCG) curves of PP-S0, PP-S1, and PP-S3 tested at 80 °C and at 95 °C in non-chlorinated water (0 mg/L free chlorine) and in chlorinated water (5 mg/L free chlorine).

To investigate and study local aging effects in chlorinated water via fatigue crack growth (FCG) experiments at predefined temperatures, it is essential to have a reference state for comparison of such FCG curves. These reference states for the test conditions were defined in terms of equivalent test temperatures but non-chlorinated water as the liquid environment. Hence, Figure 5 depicts and compares the FCG curves for the three PP grades tested at 80 °C and 95 °C in non-chlorinated water (0 mg/L free chlorine) and in chlorinated water (5 mg/L free chlorine). From these FCG curves, three essential findings may be deduced. First, enhancing the test temperature from 80 °C to 95 °C in all materials and in both liquids (non-chlorinated water and chlorinated water) leads to a significant increase in FCG rates over the entire stress intensity factor range investigated. Second, and again for all three PP grades, the chlorinated water environment at a given temperature also results in a significant enhancement of FCG rates. In other words, the chlorinated water environment clearly leads to a deterioration of the FCG resistance in these PP materials. Third, when comparing the FCG data generated at 80 °C with those obtained at 95 °C, the deterioration of the FCG resistance by the chlorinated water environment is noticeably magnified at the higher temperature.

**Figure 6:**
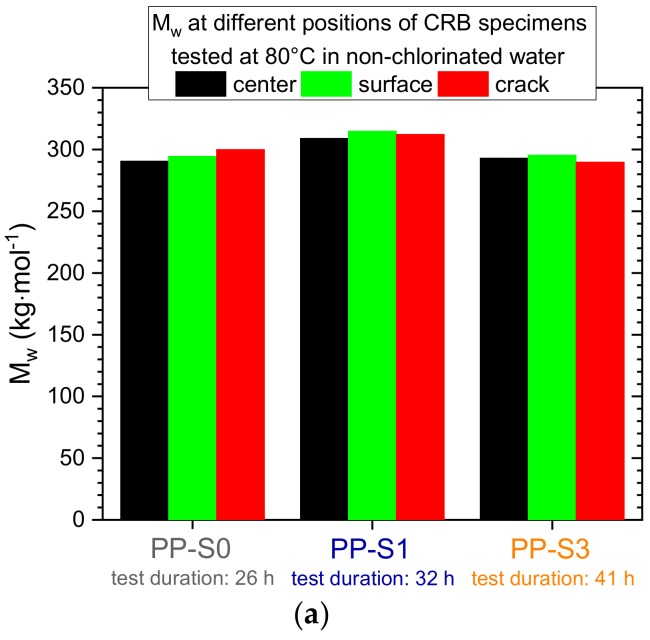

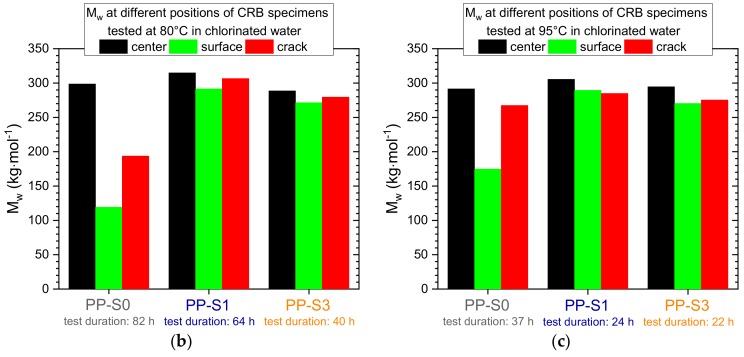
Mean weight average molar masses (*M*w) of samples taken from three different positions in CRB specimens of PP-S0, PP-S1, and PP-S3, which were tested under superimposed mechanical-environmental conditions. The fatigue crack growth tests were performed at (**a**) 80 °C in non-chlorinated water (0 mg/L free chlorine); (**b**) 80 °C in chlorinated water (5 mg/L free chlorine); and (**c**) 95 °C in chlorinated water (5 mg/L free chlorine). The total FCG test duration is also indicated.

The local aging states at the three predefined positions of the CRB specimens (center, surface, and crack as indicated in Figure 2) tested under the various conditions of test temperature and liquid environment are depicted in terms of local mean molar masses (M_w_) in Figure 6a (non-chlorinated water at 80 °C), Figure 6b (chlorinated water at 80 °C), and Figure 6c (chlorinated water at 95 °C). Starting with the data for non-chlorinated water at 80 °C depicted in Figure 6a, it may be concluded that these environmental conditions do not affect the M_w_ values to a significant degree at least within the maximum test duration of 41 h. Independent of the sample position, M_w_ values were found to range in a rather narrow window from 290 kg/mol to 315 kg/mol. Hence, it appears that essentially no molecular degradation takes place under these test conditions.

In sharp contrast, significant molecular degradation was detected for the surface and crack position of PP-S0, containing no long-term stabilization, when tested in chlorinated water at 80 °C (Figure 6b) and at 95 °C (Figure 6c). For example, when tested in chlorinated water at 80 °C, the M_w_ value at the surface position of a CRB specimen tested for 82 h dropped to 119 kg/mol, whereas the M_w_ value at the crack position was reduced to 194 kg/mol. Compared to the M_w_ value of 299 kg/mol for the center position of this CRB specimen, which was not directly exposed to the liquid environment while being tested, this corresponds to an M_w_ reduction of 60% for the surface position and 35% for the crack position. Analogous reductions, albeit less pronounced, were found for the M_w_ values of PP-S0 at the surface and crack positions when tested in chlorinated water at 95 °C for 37 h (Figure 6c). In this case, the not directly medium exposed center position exhibited an M_w_ value of 292 kg/mol, while the corresponding numbers for the surface and crack position were found to be 175 kg/mol and 267 kg/mol, respectively. In relative terms, these reductions amount to 40% and 8%, respectively. Apparently, the total test duration and, hence, the total direct exposure time of local material regimes to chlorinated water, may be the key parameter determining the degree of local aging, at the specimen surface or at the fracture surface. In other words, the higher reduction in M_w_ at the surface and crack positions for tests performed at 80 °C versus those at 95 °C are believed to be a result of the twice as long test duration from the start of the test to ultimate failure (82 h at 80 °C vs. 37 h at 95 °C). This also may help explain why the molecular degradation at the surface position is more pronounced than at the crack position. Thus, the surface position is exposed directly to the chlorinated water for the entire duration of the FCG experiment, whereas the crack position is exposed essentially only to the fraction of the total test time once crack growth initiation commences and stable crack growth takes place.

By comparison, the local alterations in the Mw values of samples taken from the surface and crack positions compared to the center position of the long-term stabilized PP grades PP-S1 and PP-S3 are much less pronounced. While this provides clear evidence of the stabilization effectivity in terms of retarding local molecular degradation under chlorinated water conditions at elevated temperatures in these latter two materials, the data do reveal some local molecular degradation even for these long-term stabilized materials. For example, comparing the crack position to the center position of failed CRB specimens of PP-S1 and PP-S3, for both materials, Mw reductions of about 3% and 7% were detected for tests in chlorinated water at 80 °C and at 95 °C, respectively. In this case, it must be kept in mind that the overall test duration to generate FCG curves for PP-S1 and PP-S3 was shorter than the test time for PP-S0. Moreover, when focusing on the ultra-high molar mass fraction of the molecular mass distribution with molar masses above 3000 kg/mol, the analysis of the local aging states of surface and crack samples of tests in chlorinated water at 80 °C and 95 °C, respectively, revealed a reduction of this ultra-high molar mass fraction by about 9% and 25%.

## 4. Discussion

Before discussing and comparing the specific results of the two test series performed, with one being defined as global aging of micro-sized specimens, and the other as local aging in CRB specimens under superimposed mechanical-environmental conditions, it is worthwhile to emphasize some key differences in essential parameters. In the global aging test series, micro-sized specimens with a thickness of 0.1 mm were pre-exposed to chlorinated water at 60 °C without any mechanical loads up to 750 h. The subsequent mechanical tests were then performed under monotonic tensile conditions in air at 23 °C. In contrast, for the local aging test series, fatigue crack growth experiments were performed with CRB specimens with a diameter of 14 mm in non-chlorinated water and chlorinated water at elevated temperatures of 80 °C and 95 °C. In this case, the test duration and, hence, the exposure time of the material to the liquid environment corresponded to the time to failure of these specimens under the various test conditions. This test duration turned out to range from 22 h to 82 h, which implies a much shorter exposure time to the liquid environment. Due to these differences, any cross-correlations between the results of the two test series are quite limited.

### 4.1. Effect of Chlorinated Water on Global Aging Behavior of Micro-Sized Specimens

**Figure 7:**
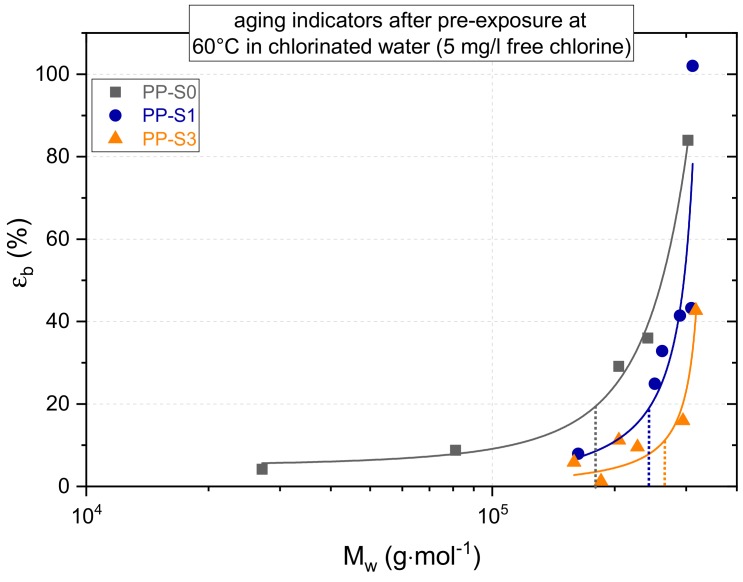
Dependence of strain at break values (ε_b_) of the three aged PP grades on the mean molar mass (M_w_) of these materials in the aged state with indications on the vertex (dotted line) of the fit curvature representing the characteristic embrittlement mean molar mass of the materials (PP-S0: 180 kg/mol), PP-S1: 243 kg/mol, PP-S3: 265 kg/mol).

The dependence of the strain at break (ε_b_) on the mean weight average molar mass (M_w_) is shown in Figure 5 for unexposed and pre-exposed samples of the three PP grades. Referring to a discussion in one of our previous papers [12], in this diagram, the results are plotted in a linear-log scale since this form of data representation allows for a better definition of characteristic mean molar mass values. Below these characteristic mean molar mass values the materials rapidly become brittle as the strain at break values of aged materials approach the yield strains (i.e., characteristic embrittlement mean molar mass, M_w,ch_). Hence, the data in Figure 7 are depicted together with exponential fit functions, from which the M_w,ch_ values may be determined as the vertex of the fit curvature. These M_w,ch_ values are indicated in Figure 7 as dotted lines and range from 180 kg/mol for PP-S0 to 243 kg/mol for PP-S1 to 265 kg/mol for PP-S3. Considering these numbers, two aspects deserve mentioning. First, the M_w,ch_ values determined here for PP homopolymers of different stabilization and ranging from 180 to 265 kg/mol are below the value of about 300 kg/mol reported for PP random copolymers [12,41,51]. Second, the finding of different M_w,ch_ values for the three PP formulations with an identical base polymer investigated in this case, is most likely related to the fact that the stabilizer system used does not only control the degradation kinetics but also has a pronounced influence on the molecular mass distribution (MMD) of the aged polymer. In other words, the stabilizer system used has a pronounced influence on the degree of broadening of the MMD up to the development of bimodal and multimodal MMD in aged polymers. This latter argument, while principally plausible, is consistent with the findings on the MMD evolution for these three materials in Figure 3.

**Figure 8:**
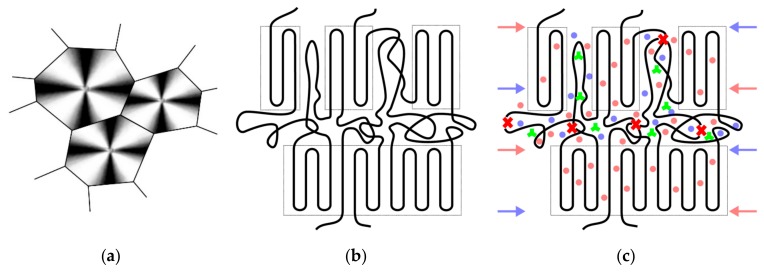
Schematic of key features of the inner material structure of semi-crystalline polymers and the interaction with environmental conditions, (**a**) spherulite structure, (**b**) stacked lamellar structure with inter-spherulitic and inter-lamellar amorphous domains with tie molecules and entanglements, and (**c**) stacked lamellar structure with stabilizers in the amorphous regions (green stars) and interaction with environmental conditions (temperature: red arrows and dots, liquid: blue arrows and dots) leading to aging-induced chain scission (red crosses) in the amorphous regime.

While there may be numerous reasons for different stabilizer systems to cause alterations in the shape of the MMD upon material global aging (i.e., various degrees of MMD broadening and multimodality), an important aspect to consider is that aging in semi-crystalline polymers is believed to occur predominantly in the amorphous regimes between spherulites and crystal lamellae [12,43]. These regimes, however, represent also the areas, where stabilizers are preferentially situated and where environmental liquids such as chlorinated water penetrate into the polymer [12,43]. Taken together, these three aspects also explain why and, to some degree, how the aging kinetics is influenced by the presence of stabilizers and the type and concentration of stabilizer systems used. In terms of chemical polymer aging and molecular degradation, it is especially critical if the inter-spherulitic and inter-lamellar tie molecule and entanglement density is affected. This inter-spherulitic and inter-lamellar molecular degradation mechanism along with some of the micro-structural features and the presence of stabilizers along with the present environmental conditions (temperature and aggressive liquids) is shown in Figure 8.

### 4.2. Effect of Chlorinated Water on the Fatigue Crack Growth Resistance and Local Aging Behavior in CRB Specimens

Referring back to Figure 5 and Figure 6, the test duration and the investigated crack growth rate regime, have a pronounced effect on local aging. Hence, in Table 2, the deterioration factors determined as the ratio between the environmental-dependent crack growth rates at a given stress intensity factor range (ΔK = 0.3 MPa*m^0.5^) are depicted for the two test temperatures at 80 °C and 95 °C, comparing non-chlorinated water (reference environment with a deterioration factor of 1) with chlorinated water. With deterioration factors ranging from 4 to 9 for 80 °C and from 32 to 54 for 95 °C, significantly higher values were found at 95 °C, which indicates that the more oxidative environment (chlorinated water) is more aggressive at higher temperatures. Comparing the deterioration factors for chlorinated water at a given temperature, one must be cautious to not over interpret the data, since the reproducibility range for crack growth rates at a given ΔK value is also typically found to be in the order of a factor of 2. At both temperatures, the difference in the deterioration factors between PP-S1 and the other two formulations is still within this range. Yet, it should also be mentioned that moving from the accelerated laboratory test conditions with test durations of less than 100 h to real service conditions with exposure times of several years may even greatly enhance these local aging effects at crack tips in these materials.

**Figure 9:**
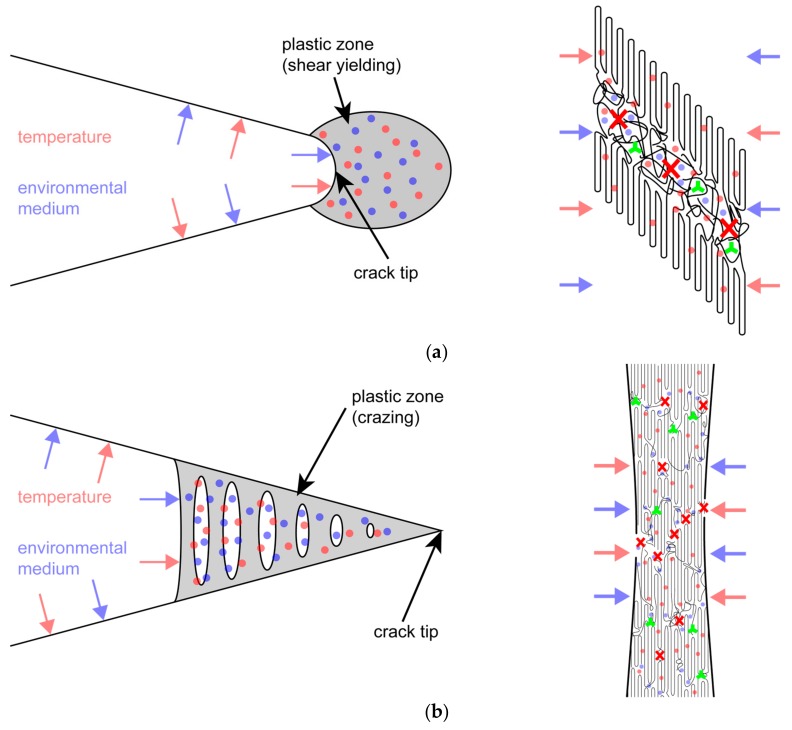
Schematic illustration of the crack tip region under superimposed mechanical-environmental loading (red arrows and dots representing temperature, blue arrows and dots representing environmental liquid, green stars representing stabilizer molecules, red crosses representing molecular chain scission). (**a**) Plastic deformation by shear yielding with the crack tip shear yield zone (**left**) and irreversible shear deformation of semi-crystalline lamellar structure (**right**). (**b**) Plastic deformation by crazing indicating the wedge-shaped fibril/void structure of a crack tip craze (**left**) and a highly stretched semi-crystalline craze fibril (**right**).

While the above data provide clear evidence on the deteriorating effects of the chlorinated water environment on local crack tip aging, the micro and molecular mechanisms believed to cause these local crack tip aging effects are not so clear. A schematic illustration of the main mechanisms believed to be involved is provided in Figure 9 for the two primary crack tip plastic zone formation mechanisms of shear yielding (Figure 9a) and crazing (Figure 9b). According to the hypothesis of local crack tip aging [32,33], the superposition of high mechanical stresses and an oxidative environmental medium (e.g., gaseous or liquid) at an elevated temperature may greatly enhance any chemical aging reactions immediately in the crack tip region [12,15,32,33,34,35,36]. For plastic deformation by shear yielding (Figure 9a), a liquid environmental medium may accelerate local aging by the following mechanisms. Facilitated by the enhanced free volume of the mechanically stressed material, the liquid may penetrate and diffuse more easily into the shear yield zone via the amorphous domains. The liquid may then act to leach out some of the stabilizers in the shear yield zone, on the one hand, and simultaneously may cause enhanced stabilizer consumption within the shear yield zone. Both mechanisms will accelerate chemical aging in the crack tip shear yield zone, which leads to faster crack growth rates, particularly when inter-lamellar and inter-spherulitic tie molecules and entanglements are affected by molecular degradation. For crack tip plastic deformation by crazing (Figure 9b), analogous mechanisms are envisaged, which are reinforced by two additional factors. First, the fibril/void network of a craze together with the larger material surface to volume ratio allows for a much better access of the liquid, which accelerates liquid penetration and diffusion and, hence, stabilizer leakage and consumption. Second, the stretch ratio of craze fibrils is typically higher than the one in shear zones [52], which represents a more critical deformation state. This may also enhance the oxidation-enhanced cleavage of inter-lamellar and inter-spherulitic tie molecules and entanglements. While the above failure mechanisms and processes are plausible and may serve as phenomenological explanations of the observed effects under superimposed environmental loading of fatigue crack growth experiments, further research will need to be performed to corroborate these conclusions and to elucidate further details of the stress-environment-material deformation interactions.

## 5. Summary and Conclusions

The effect of hot chlorinated water on aging and fatigue crack growth (FCG) resistance was investigated for three polypropylene (PP) grades with different stabilizer systems. As reference material, a commercially available PP homopolymer (PP-S0), containing a processing stabilizer without long-term stabilization, was used. For the other two PP grades, PP-S0 was additionally stabilized with a primary phenolic antioxidant (PP-S1) and with both a primary phenolic antioxidant and a hindered amine stabilizer (PP-S3), respectively.

Micro-sized specimens were pre-exposed to chlorinated water with a chlorine content of 5 mg/L and a temperature of 60 °C for up to 750 h to monitor the influence of hot chlorinated water on global aging. Over the entire exposure period, significant aging was detected via a continuous decrease in the stabilizer content, the oxidation induction temperature, the mean weight average molar mass, and the values for strain at break. Overall, in terms of aging resistance and ultimate mechanical performance, PP-S1 was found to outperform the other two material formulations, at least for the investigated exposure conditions (i.e., 60 °C and 5 mg/L free chlorine) and test conditions (i.e., short-term tensile tests at room temperature following environmental exposure). Clearly, while the addition of a primary phenolic antioxidant in PP-S1 had a pronounced positive effect on decelerating the overall kinetics of global aging, for PP-S3 containing a primary phenolic antioxidant together with a hindered amine stabilizer, an antagonistic effect with accelerated aging was found. The characteristic embrittlement mean molar masses M_w,ch_ of the materials, when the strain at break of aged materials approaches the yield strain, were determined to range from 180 kg/mol to 265 kg/mol, with PP-S0 exhibiting the lowest and PP-S3 exhibiting the highest value. Apparently, the stabilizer systems do not only control the degradation kinetics but also affect the molar mass distribution (MMD) of the aged polymer via a pronounced influence on the degree of broadening of the MMD, for PP-S3 even inducing the development of bimodal and multimodal MMD in an advanced aging state.

Superimposed mechanical-environmental cyclic tests of cracked round bar (CRB) specimens with the three PP grades in non-chlorinated (0 mg/L free chlorine) and chlorinated (5 mg/L free chlorine) water at 80 °C and 95 °C provided clear evidence of enhanced local crack tip aging due to the chlorinated water environment. This conclusion is not only supported by the enhanced crack growth rates determined in chlorinated water experiments for given stress intensity factor range values for all three materials at both test temperatures. It has also been validated by molar mass measurements of material samples taken from various positions of the tested CRB specimens including samples taken from the stable crack growth region of the specimen fracture surface. The enhanced tendency of local crack tip aging is related to the material microstructure of the highly stressed and plastically deformed region at the crack tip, which allows for better access of the oxidizing liquid environment into the amorphous domains of the crack tip plastic zone and the detrimental chain scission effect upon inter-lamellar and inter-spherulitic tie molecules and entanglements. As expected, due to the general acceleration of molecular degradation at higher temperatures in oxidative environments, a temperature increase from 80 °C to 95 °C also resulted in a significant enhancement of chlorine-induced local crack tip aging in all three material grades. Lastly, and to some extent surprisingly, in the chlorinated water environment under these superimposed mechanical-environmental conditions, the performance differences between the three PP grades nearly vanished.

## Figures and Tables

**Table 1:** Material designation and stabilizer content.

Material Designation	Primary Phenolic Antioxidant	Hindered Amine Stabilizer
PP-S0	-	-
PP-S1	0.3 m%	-
PP-S3	0.3 m%	0.3 m%

**Table 2:** Deterioration factors for the crack growth rate at ΔK = 0.3 MPa·m^0.5^ for the three materials investigated at 80 °C and 95 °C in non-chlorinated and chlorinated water.

Material	80 °C		95 °C	
	Non-Chlorinated Water	Chlorinated Water	Non-Chlorinated Water	Chlorinated Water
PP-S0	1	9	1	32
PP-S1	1	4	1	54
PP-S3	1	6	1	32

## References

[1] ISO (2013). ISO/TC 138/SC 2 Plastics pipes and fittings for water supplies. ISO 15874-1:2013 Plastics Piping Systems for Hot and Cold Water Installations—Polypropylene (PP)—Part 1: General.

[2] F17 Committee (2007). Specification for Pressure-Rated Polypropylene (PP) Piping Systems.

[3] Gahleitner M., Paulik C. (2017). Polypropylene and Other Polyolefins. Brydson’s Plastics Materials.

[4] Grabmann M.K., Wallner G.M., Grabmayer K., Nitsche D., Lang R.W. (2018). Aging behavior and lifetime assessment of polyolefin liner materials for seasonal heat storage using micro-specimen. Sol. Energy.

[5] Grabmann M.K., Wallner G.M., Ramschak T., Buchinger R., Lang R.W., Martínez V., González J. (2016). Global Aging and Lifetime Prediction of Polymeric Materials for Solar Thermal Systems-Part 1: Polypropylene Absorbers for Pumped Systems. EuroSun2016, Proceedings of the EuroSun2016, Palma de Mallorca, Spain, 11–14 October 2016.

[6] Grabmann M.K., Wallner G.M., Ramschak T., Ziegler G., Lang R.W., Martínez V., González J. (2016). Global Aging and Lifetime Prediction of Polymeric Materials for Solar Thermal Systems-Part 2: Polyamide 66 Glass Fiber Reinforced Absorbers for Integrated Storage Collectors. EuroSun2016, Proceedings of the EuroSun2016, Palma de Mallorca, Spain, 11–14 October 2016.

[7] Wallner G.M., Povacz M., Hausner R., Lang R.W. (2016). Lifetime modeling of polypropylene absorber materials for overheating protected hot water collectors. Sol. Energy.

[8] Deborde M., Von Gunten U.R.S. (2008). Reactions of chlorine with inorganic and organic compounds during water treatment-Kinetics and mechanisms: A critical review. Water Res..

[9] World Chlorine Council Drinking Water Chlorination: World Chlorine Council Position Paper 2008. www.worldchlorine.org.

[10] World Health Organization (2011). Guidelines for Drinking-Water Quality.

[11] Bartram J., Chartier Y., Lee J.V., Pond K., Surman-Lee S. (2007). Legionella and the Prevention of Legionellosis.

[12] Fischer J., Freudenthaler P.J., Lang R.W., Buchberger W., Mantell S.C. (2019). Chlorinated Water Induced Aging of Pipe Grade Polypropylene Random Copolymers. Polymers.

[13] Fischer J., Mantell S.C., Bradler P.R., Wallner G.M., Lang R.W. (2019). Effect of aging in hot chlorinated water on the mechanical behavior of polypropylene grades differing in their stabilizer systems. Mater. Today Proc..

[14] Fischer J., Bradler P.R., Lang R.W. Fatigue crack growth testing in chlorinated water at elevated temperatures-test equipment. Proceedings of the Plastic Pipes XIX.

[15] Fischer J., Bradler P.R., Lang R.W. (2018). Test equipment for fatigue crack growth testing of polymeric materials in chlorinated water at different temperatures. Eng. Fract. Mech..

[16] Fischer J., Eckerstorfer M., Bradler P.R., Wallner G.M., Lang R.W. Investigation of the effect of stabilizer system, medium and temperature on the fatigue crack growth resistance of polypropylene for a proper material selection. Proceedings of the ANTEC 2018.

[17] Fischer J., Freudenthaler P.J., Bradler P.R., Lang R.W., Mantell S.C. Effect of beta-nucleation on aging and crack growth resistance of polypropylene exposed to chlorinated water. Proceedings of the Plastic Pipes XIX.

[18] Fischer J., Bradler P.R., Akhras M.H., Wallner G.M., Lang R.W. (2018). Influence of Hot Chlorinated Water and Stabilizer Package on the Fatigue Crack Growth Resistance of Glass Fiber Reinforced Polyamide. Polymers.

[19] Fischer J., Bradler P.R., Lang R.W., Wallner G.M. Fatigue crack growth resistance of polypropylene in chlorinated water at different temperatures. Proceedings of the Plastic Pipes XVIII.

[20] Fischer J., Mantell S.C., Bradler P.R., Wallner G.M., Lang R.W., Romero M., Mugnier D., Renné D., Guthrie K., Griffiths S. (2017). Effect of Aging in Hot Chlorinated Water on the Mechanical Behavior of Polypropylene for Solar-Thermal Applications. SWC2017/SHC2017, Proceedings of the ISES Solar World Conference 2017 and the IEA SHC Solar Heating and Cooling Conference for Buildings and Industry 2017, Abu Dhabi, United Arab Emirates, 29 October–2 November 2017.

[21] Hassinen J., Lundbaeck M., Ifwarson M., Gedde U. (2004). Deterioration of polyethylene pipes exposed to chlorinated water. Polym. Degrad. Stab..

[22] Majewski K., Cosgriff E., Mantell S., Bhattacharya M. Fracture Properties of HDPE Exposed to Chlorinated Water. Proceedings of the ANTEC 2018.

[23] F17 Committee (2014). Test Method for Evaluating the Oxidative Resistance of Polyethylene (PE) Pipe to Chlorinated Water.

[24] F17 Committee (2015). Test Method for Evaluating the Oxidative Resistance of Crosslinked Polyethylene (PEX) Tubing and Systems to Hot Chlorinated Water.

[25] Yu W., Reitberger T., Hjertberg T., Oderkerk J., Costa F.R., Gedde U.W. (2012). Antioxidant consumption in squalane and polyethylene exposed to chlorinated aqueous media. Polym. Degrad. Stab..

[26] Castillo Montes J., Cadoux D., Creus J., Touzain S., Gaudichet-Maurin E., Correc O. (2012). Ageing of polyethylene at raised temperature in contact with chlorinated sanitary hot water. Part I—Chemical aspects. Polym. Degrad. Stab..

[27] Vibien P., Couch J., Oliphant K., Zhou W., Zhang B., Chudnovsky A. Assessing material perfomance in chlorinated potable water applications. Proceedings of the Plastic Pipes XI.

[28] Colin X., Audouin L., Verdu J., Rozental-Evesque M., Rabaud B., Martin F., Bourgine F. (2009). Aging of polyethylene pipes transporting drinking water disinfected by chlorine dioxide. I. Chemical aspects. Polym. Eng. Sci..

[29] Colin X., Audouin L., Verdu J., Rozental-Evesque M., Rabaud B., Martin F., Bourgine F. (2009). Aging of polyethylene pipes transporting drinking water disinfected by chlorine dioxide. Part II-Lifetime prediction. Polym. Eng. Sci..

[30] Damodaran S., Schuster T., Rode K., Sanoria A., Brüll R., Wenzel M., Bastian M. (2015). Monitoring the effect of chlorine on the ageing of polypropylene pipes by infrared microscopy. Polym. Degrad. Stab..

[31] Seidler D. (2012). Aus Schaden klug werden. Kunststoffe.

[32] Lang R.W., Stern A., Doerner G. (1997). Applicability and limitations of current lifetime prediction models for thermoplastics pipes under internal pressure. Angew. Makromol. Chemie Appl. Macromol. Chem. Phys..

[33] Lang R.W., Pinter G., Balika W. (2005). Konzept zur Nachweisführung für Nutzungsdauer und Sicherheit von PE-Druckrohren bei beliebiger Einbausituation. 3R International.

[34] Pinter G., Haager M., Wolf C., Lang R.W. (2004). Thermo-Oxidative Degradation during Creep Crack Growth of PE-HD Grades as Assessed by FT-IR Spectroscopy. Macromol. Symp..

[35] Pinter G., Duretek I., Aust N., Lang R.W. (2002). Characterisation of the thermo-oxidative degradation of polyethylene pipes by chromatographical, rheological and thermo-analytical methods. Macromol. Symp..

[36] Pinter G., Lang R.W. (2003). Effect of stabilization on creep crack growth in high-density polyethylene. J. Appl. Polym. Sci..

[37] Zweifel H. (1993). Plastics Additives Handbook.

[38] Yu W., Reitberger T., Hjertberg T., Oderkerk J., Costa F.R., Englund V., Gedde U.W. (2015). Chlorine dioxide resistance of different phenolic antioxidants in polyethylene. Polym. Degrad. Stab..

[39] Grabmayer K., Beißmann S., Wallner G.M., Nitsche D., Schnetzinger K., Buchberger W., Schobermayr H., Lang R.W. (2015). Characterization of the influence of specimen thickness on the aging behavior of a polypropylene based model compound. Polym. Degrad. Stab..

[40] Grabmann M., Wallner G., Grabmayer K., Buchberger W., Nitsche D. (2018). Effect of thickness and temperature on the global aging behavior of polypropylene random copolymers for seasonal thermal energy storages. Sol. Energy.

[41] Wallner G.M., Grabmann M.K., Klocker C., Buchberger W., Nitsche D. (2018). Effect of carbon nanotubes on the global aging behavior of β-nucleated polypropylene random copolymers for absorbers of solar-thermal collectors. Sol. Energy.

[42] Grabmann M.K., Wallner G.M., Maringer L., Buchberger W., Nitsche D. (2019). Hot air aging behavior of polypropylene random copolymers. J. Appl. Polym. Sci..

[43] Maringer L., Grabmann M., Muik M., Nitsche D., Romanin C., Wallner G., Buchberger W. (2017). Investigations on the distribution of polymer additives in polypropylene using confocal fluorescence microscopy. Int. J. Polym. Anal. Charact..

[44] Grabmayer K. (2014). Polyolefin-Based Lining Materials for Hot Water Heat Storages Development of Accelerated Aging Characterization Methods and Screening of Novel Compounds. Ph.D. Dissertation.

[45] ISO (2015). ISO/TC 138/SC 5 General properties of pipes, fittings and valves of plastic materials and their accessories—methods and basic specifications. ISO 18489:2015 Polyethylene (PE) Materials for Piping Systems—Determination of Resistance to Slow Crack Growth under Cyclic Loading—Cracked Round Bar Test Method.

[46] Cosgriff E., Mantell S. Method for degrading polyethylene sheet samples in an oxidative environment. Proceedings of the ANTEC 2017.

[47] ISO (2018). ISO/TC 61/SC 5 Physical-chemical properties. ISO 11357-3:2018 Plastics—Differential Scanning Calorimetry (DSC)—Part 3: Determination of Temperature and Enthalpy of Melting and Crystallization.

[48] Beißmann S., Reisinger M., Grabmayer K., Wallner G., Nitsche D., Buchberger W. (2014). Analytical evaluation of the performance of stabilization systems for polyolefinic materials. Part I: Interactions between hindered amine light stabilizers and phenolic antioxidants. Polym. Degrad. Stab..

[49] Ohkatsu Y., Fujiwara T. (2007). Interaction between Nitroxide of Hindered Amine Light Stabilizers and Phenol. J. Jpn. Petrol. Inst..

[50] Ohkatsu Y. (2008). Search for Unified Action Mechanism of Hindered Amine Light Stabilizers. J. Jpn. Petrol. Inst..

[51] Fayolle B., Audouin L., Verdu J. (2004). A critical molar mass separating the ductile and brittle regimes as revealed by thermal oxidation in polypropylene. Polymer.

[52] Donald A.M., Kramer E.J. (1982). The competition between shear deformation and crazing in glassy polymers. J. Mater. Sci..

